# Bladder paraganglioma in pregnancy with a successful perinatal outcome: a case report

**DOI:** 10.1093/bjrcr/uaaf026

**Published:** 2025-04-29

**Authors:** Roy Teng, Joshua Silverman, Scott McClintock, Patricia Deonarine

**Affiliations:** Urology Department, Gold Coast University Hospital, Gold Coast 4215, Australia; Urology Department, Gold Coast University Hospital, Gold Coast 4215, Australia; Urology Department, Gold Coast University Hospital, Gold Coast 4215, Australia; Radiology Department, Gold Coast University Hospital, Gold Coast 4215, Australia

**Keywords:** paraganglioma, pregnancy, bladder, cystectomy

## Abstract

Paragangliomas (PGLs) during pregnancy is an uncommon neuroendocrine tumour that is associated with increased maternal and foetal morbidity and mortality. Furthermore, it is even rarer for these to be located within the urinary bladder, with a prevalence of <0.1% of all bladder tumours. This case report details a 29-year-old female who presented with pre-syncope, headache, and palpitations during voiding. Ultrasound and magnetic resonance imaging of the pelvis revealed a mass in her bladder, and biochemical workup demonstrated elevated plasma normetanephrine levels and a positive clonidine suppression test. Surgical resection and histopathology of the mass were consistent with PGL. Post-operatively, the patient was normotensive, her normetadrenaline levels normalized and she was discharged 3 days after the operation. She progressed through the remaining pregnancy without any significant complications and delivered a healthy baby at full term. This case depicts the rare nature of PGLs in pregnancy and the importance of antenatal imaging combined with a multidisciplinary approach for a successful pregnancy outcome.

## Case report

**Figure 1.**
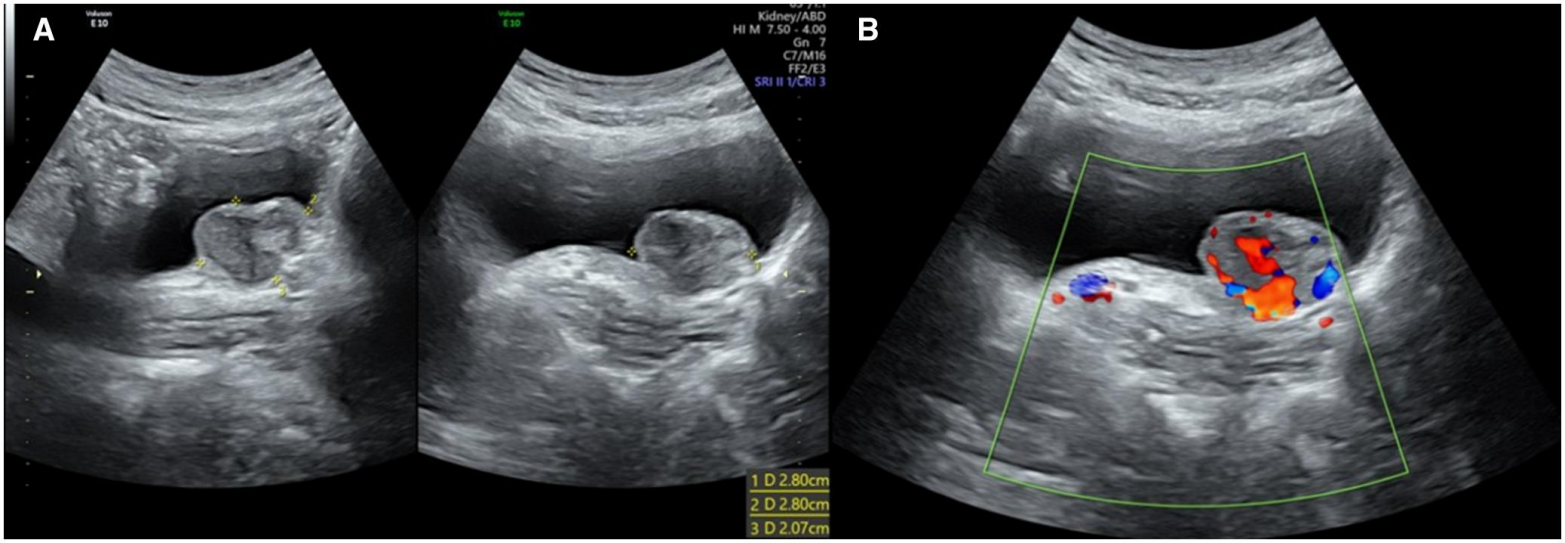
(A) Ultrasound image of the bladder showing the paraganglioma with corresponding size of 2.80 × 2.80 cm. (B) Colour doppler of the mass demonstrates increased vascularity of the lesion. Images were taken from the Gold Coast PACS system.

A 29-year-old G1P0 presented with a history of paroxysmal headaches, palpitations, flushing, and pre-syncope whilst voiding at gestational age of 10 weeks. Her heart rate varied between 66 and 75 bpm with a normal blood pressure at 122/88 mmHg. She denied any significant cardiac or other medical history and did not take any regular medications. On the initial dating ultrasound, there was a 28 mm highly vascular and hypoechoic rounded bladder wall lesion found to the left of the urethra (Figure 1). Laboratory investigations including full blood count, electrolytes, liver function test, and renal function test were normal. She had an elevated supine plasma normetadrenaline at 3120 µmol/L (120-600 µmol/L), Chromogranin A was above the normal range at 104 µg/L (20-102 µg/L) and her 24-h urinary normetadrenaline was elevated at 9.5 µmol/L (<2.3 µmol/L).

**Figure 2.**
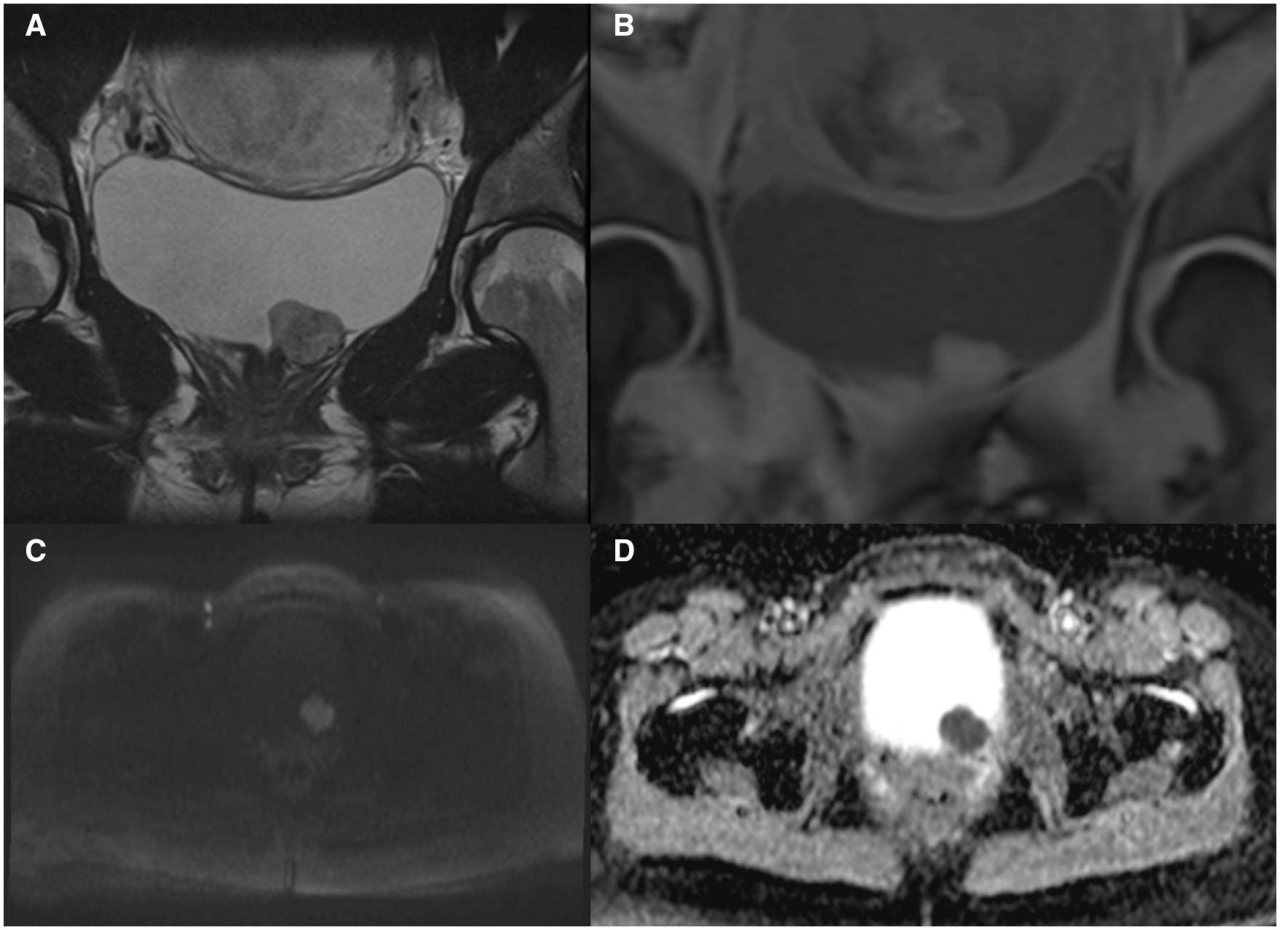
(A) A T2 image showing a well-defined lesion from the anterior bladder wall with homogenous T2 intermediate signal changes and hypointense foci representing internal vascularity, with (B) corresponding to the coronal view of the mass in T1 with mild hyperintensity. (C) An axial view in the DWI sequence showcasing the restricted diffusion of the mass. (D) The same lesion with hypointense signal on ADC map, further suggesting restricted diffusion. Abbreviations: ADC = apparent diffusion coefficient; DWI = diffusion-weighted image. Images were taken from the Gold Coast PACS system.

A cystoscopy was performed and visualized a submucosal lesion between the left ureteric orifice and the urethra. An urgent magnetic resonance imaging (MRI) of her pelvis was organized which demonstrated a 33 × 16 × 25 mm homogenously T2 intermediate, mildly T1 fat-saturated hyperintense signal mass arising from the bladder wall on the lateral aspect of the proximal urethra. Internal T2 serpiginous hypointense areas consistent with internal vascularity were noted. There was marked restricted diffusion consistent with a highly cellular lesion (Figure 2). No post-contrast sequences were acquired given the patient was scanned in pregnancy.

**Figure 3.**
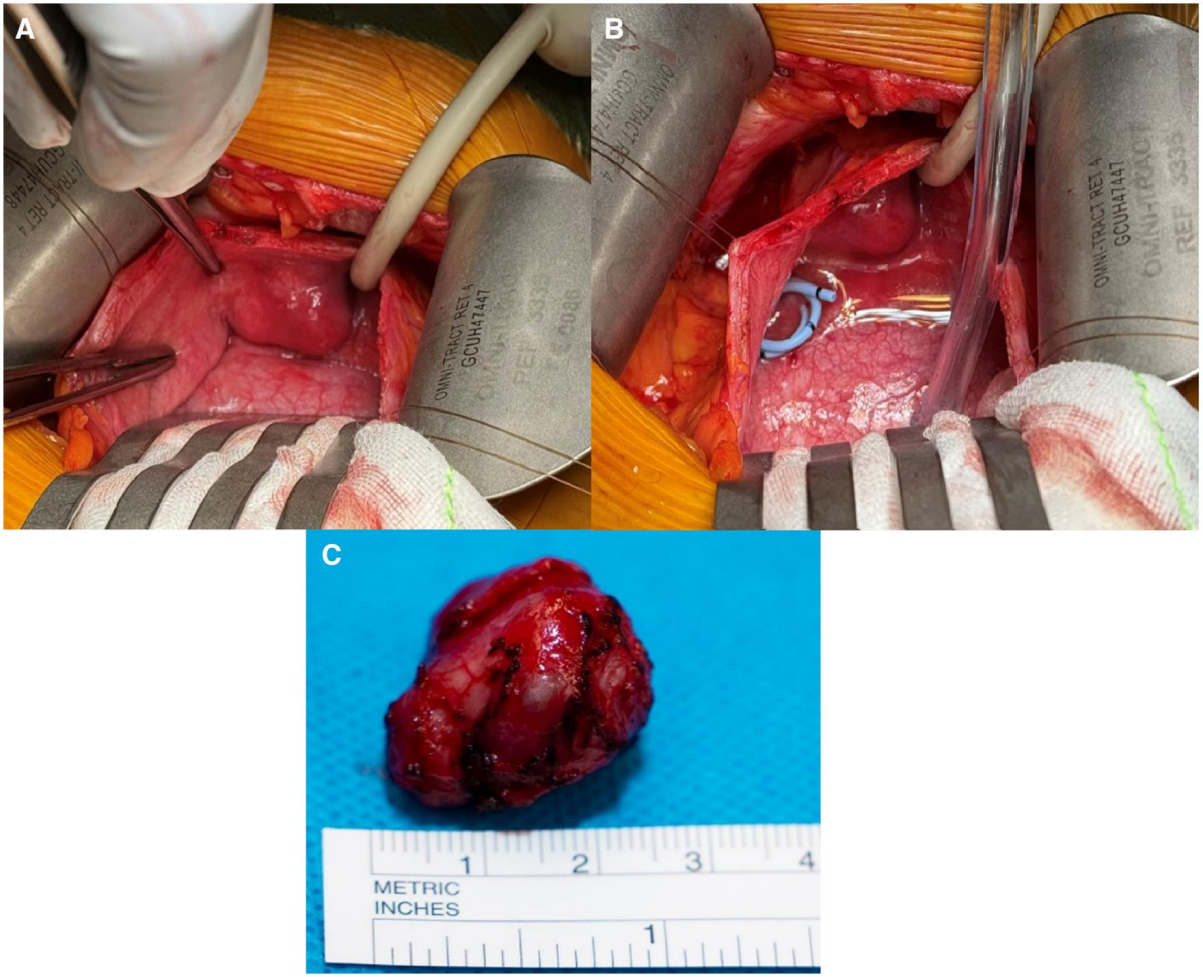
(A) The superior view from patient’s perspective of the lesion within the urinary bladder. Note the indwelling urinary catheter to the right of the lesion. (B) Similar perspective, with a double-J ureteric stent indicating the ureteric orifice location in relation to the bladder mass. (C) The surgical specimen measuring 2.5 cm, showing a well-circumscribed lesion. Images were taken from the Gold Coast PACS system.

**Figure 4.**
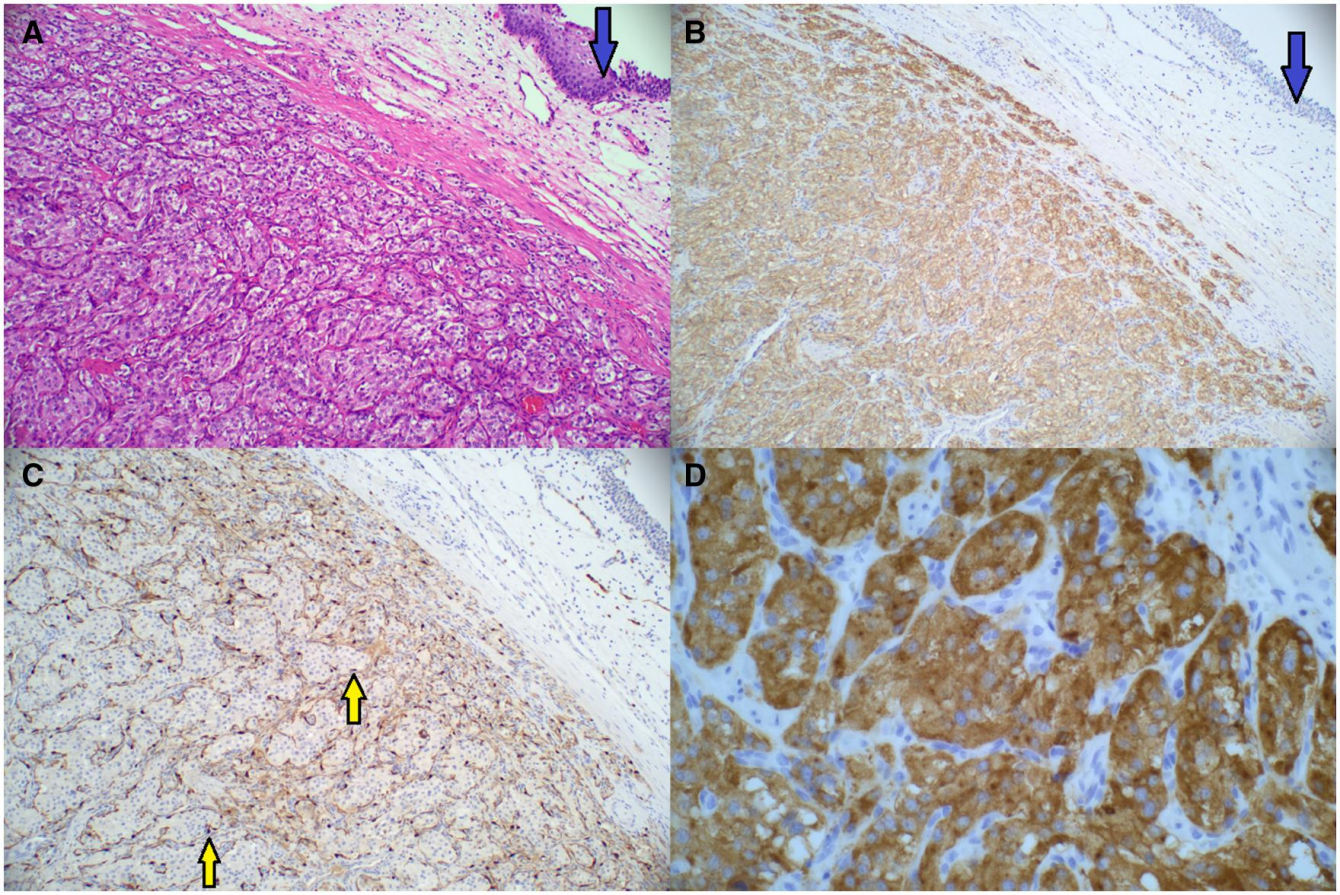
(A) Haematoxylin and eosin stained slide showing the paraganglioma with nested pattern on left side of the stain. (B) CD56 positive staining. Normal urothelium indicated by the blue arrow. (C) Positive S100 protein staining highlighting the sustentacular cells with nuclei (yellow arrow) stained brown around the nests. (D) Histopathology slide showing positive Chromogranin staining. Images were taken from the Gold Coast PACS system.

A multidisciplinary opinion between the Urology, Radiology, Obstetric medicine, Endocrinology, and Anaesthetic departments was sought with the consensus for the patient to undergo surgical resection with a stringent peri-operative blood pressure management. She was commenced on Prazosin, an α-adrenoreceptor blocker, 2 weeks prior, then switched to Propanolol, a β-adrenoreceptor blocker, 1 week before her operation with intravenous fluid loading. She underwent a partial cystectomy in her second trimester at 19 weeks. Intraoperatively, she required a low-dose noradrenaline infusion for transient hypotension, but there were no other intraoperative complications. The excised tumour was a well-circumscribed solid lesion with a covering layer of normal epithelium (Figure 3). Histopathology confirmed a paraganglioma (PGL), with immunohistochemistry showing positive staining for CD56, chromogranin and S100 protein, and negative for cytokeratin (Figure 4). Reassuringly, SDHB and SDHA gene mutation was negative.

The postoperative period was uneventful, her systolic blood pressure remained between 80 and 90 mmHg, and her remaining vital signs were within normal ranges. Her post-operative full blood count, electrolytes, renal and liver function were also normal and the patient was discharged after a 3-day admission. In the follow-up appointments, the patient’s symptoms of palpitations, flushing, and pre-syncope whilst voiding had resolved and a post-partum DOTATE positron emission tomography (PET) scan did not illustrate any residual or metastatic disease. Her normetadrenaline plasma levels normalized to 348 µmol/L 1 month after her resection. There were no other complications throughout the rest of her pregnancy. During her labour, there was concern for foetal distress on the cardiotocography, but she was able to vaginally deliver a healthy term child in the same hospital where she underwent the surgical resection.

## Discussion

PGLs are neuroendocrine neoplasms that occur outside the adrenal gland and hence are also known as extra-adrenal pheochromocytomas. These arise from chromaffin tissue that has failed to involute and are generally found in paravertebral and para-aortic autonomic nervous system.^1^ The most common region is near the inferior mesenteric artery origin, also known as organs of Zuckerkandl. Bladder PGLs represent <1% of all PGLs, and PGLs associated with pregnancy are incredibly rare, between 0.001% and 0.007% of all pregnancies.^2^

Clinical features of bladder PGLs vary from paroxysmal hypertension, headaches, diaphoresis, palpitations, and micturition syncope.^3^ New onset hypertension prior to 20 weeks gestation is more suggestive of a phaeochromocytoma or PGL rather than pre-eclampsia. Once PGL is suspected, plasma metanephrine and 24-h urinary metanephrines and catecholamines are the suggested biochemical tests. A supine urine collection is also useful if there are indeterminate results.^4^ Chromogranin A is another sensitive marker for neuroendocrine tumours and is also elevated in the presence of PGLs.

The imaging modalities that can be used to evaluate PGLs are primarily contrast-enhanced computed tomography (CT) or MRI. Ultrasound can be a useful initial imaging modality, and the majority of bladder PGLs were hypoechoic with increased Doppler flow due to the typical high vascularity. Due to the low sensitivity and non-specific appearances, ultrasound is not recommended in the evaluation of suspected PGLs, unless in certain populations (ie, paediatric or pregnant women).^5^ Therefore, CT is usually the first anatomic imaging modality utilized for PGL evaluation. Advantages of CT imaging are the short scanning time and higher spatial resolution compared to MRI. It demonstrates a well-delineated hyperdense lesion with marked post-contrast enhancement, again showcasing the rich blood supply.^5^ However, MRI is often the first-choice technique due to superior tissue characterization and lack of radiation. The typical MR findings are mildly hyperintense T1 images with significant hyperintensity on T2-weighted images and restricted diffusion on diffusion-weighted images and apparent diffusion coefficient map in comparison to the adjacent musculature. A “salt and pepper” appearance can also be seen in bladder PGLs whereby the “pepper” aspect reflects low signal flow voids and the “salt” component represents hyperintense foci due to microhaemorrhages or slow blood flow.^3^^,^^5^^,^^6^ Functional imaging techniques such as radiolabelled metaiodobenzylguanidine are useful in differentiating functional from non-functional PGLs. It utilizes a norepinephrine analogue which is actively taken up in most phaeochromocytomas and PGLs. This imaging technique can be beneficial for detecting multiple primary tumours or metastatic disease; however, it has a relatively lower sensitivity at 85%.^7^ Other nuclear tracers such as ^68^Ga-DOTATE and ^18^F-FDG which are somatostatin and glucose analogues respectively, are useful in detecting metastatic disease and complement anatomical imaging for both staging and localization purposes. Due to the PGLs relative rarity, it can often be mistaken for other tumours on imaging characteristics alone. Based on the T2 intermediate signal, the extent of restricted diffusion and site of origin stemming from the submucosa, the radiological differential diagnoses of this highly cellular lesion include lymphoma or a leiomyosarcoma.^6^ An aggressive bladder carcinoma is unlikely given the smooth normal bladder mucosa. Histologically, the presence of highly cellular Zellballen pattern cells with positive neuroendocrine markers of chromogranin, CD56, S-100 protein and negative for cytokeratin (Figure 4). These features differentiate PGLs from other cancers such as urothelial, mesenchymal, and carcinoid tumours.^3^

Management of PGLs during pregnancy primarily focuses on the pre-operative pharmacological blockade of the circulating catecholamines prior to surgical management. An α-adrenergic blocker should be commenced then a β-adrenergic blocker to control the blood pressure, then tachycardia and/or arrhythmias. This should be done at least 1-3 weeks prior to any surgical treatment or delivery. Surgical resection is often considered definitive treatment, either through a transurethral resection of bladder tumour (TURBT) or partial cystectomy.^3^ TURBT has been shown to have low recurrence rates in non-functional and localized tumours, whilst less desirable in the metabolically active tumours due to the potential of resecting into the tumour and therefore higher risk of catecholamine release. There have been reports that 85%-95% of patients remain recurrence free at 36-45 months after resection with partial cystectomy.^8^ There has been a large cohort study showing that there are no significant adverse maternal or foetal outcomes when comparing vaginal with caesarean delivery.^9^

Undiagnosed and untreated PGL during pregnancy has been associated with a 27-fold increase in maternal or foetal complications. These adverse consequences are primarily mediated by catecholaminergic crises and include cardiovascular compromise, arrhythmias, pulmonary overload, intracranial haemorrhage, foetal and maternal death.^10^ Even despite the improved detection rates and obstetric and anaesthetic care, maternal fatality ranges from 4%-9% compared to 7%-14% for foetal mortality rates.^10^ In this case, the patient’s mass was detected on her antenatal dating ultrasound scan, whereby she underwent further dedicated imaging such as an MRI and then a DOTATE PET for staging once she was post-partum. This case highlights the importance of early detection of this condition, with the utilization of the best imaging techniques and biochemical testing that allows the provision of appropriate management via a multidisciplinary approach.

## Learning points

- This case highlights the significant improvement of early diagnosis, medical and surgical interventions of paragangliomas in pregnancy.
- Ultrasound can be a useful imaging modality for early identification of the bladder mass, with the most useful being an MRI and nuclear functional imaging, such as MIBG or DOTATE PET.
- Biochemical testing should also be undertaken to confirm the presence of such a rare clinical entity if there is any clinical suspicion.
- Adopting a multidisciplinary approach for perioperative management.
